# Coding strategy for surface luminance switches in the primary visual cortex of the awake monkey

**DOI:** 10.1038/s41467-021-27892-3

**Published:** 2022-01-12

**Authors:** Yi Yang, Tian Wang, Yang Li, Weifeng Dai, Guanzhong Yang, Chuanliang Han, Yujie Wu, Dajun Xing

**Affiliations:** 1 State Key Laboratory of Cognitive Neuroscience and Learning & IDG/McGovern Institute for Brain Research, Beijing Normal University, Beijing, 100875 China; 2 State Key Laboratory of Cognitive Neuroscience and Learning & IDG/McGovern Institute for Brain Research, College of Life Sciences, Beijing Normal University, Beijing, China

**Keywords:** Sensory processing, Striate cortex

## Abstract

Both surface luminance and edge contrast of an object are essential features for object identification. However, cortical processing of surface luminance remains unclear. In this study, we aim to understand how the primary visual cortex (V1) processes surface luminance information across its different layers. We report that edge-driven responses are stronger than surface-driven responses in V1 input layers, but luminance information is coded more accurately by surface responses. In V1 output layers, the advantage of edge over surface responses increased eight times and luminance information was coded more accurately at edges. Further analysis of neural dynamics shows that such substantial changes for neural responses and luminance coding are mainly due to non-local cortical inhibition in V1’s output layers. Our results suggest that non-local cortical inhibition modulates the responses elicited by the surfaces and edges of objects, and that switching the coding strategy in V1 promotes efficient coding for luminance.

## Abstract (web summary)

How brightness is encoded in the visual cortex remains incompletely understood. By recording from macaque V1, the authors revealed a switch from surface to edge encoding that is mediated by widespread inhibition in the output layers of the cortex.

## Introduction

Both surface luminance (the intensity of light reflected from an object’s surface) and edge contrast (the changes in luminance that occur at edges) provide essential information to define an object’s identity. It has been shown that the lack of either component reduces the efficiency of the visual system to recognize an object^1,2^. The neuronal recognition of object luminance in the visual cortex has been studied for decades^3–8^. However, the cortical processing of the response to surface luminance in the primary visual cortex (V1) is still under debate.

According to the textbooks, the neural response to surface luminance is largely weakened by a center-surround antagonist receptive field structure of neurons at the pre-cortical stage;^9^ and V1 neurons are assumed to respond highly to edge contrast and react poorly to spatially uniform/low-spatial frequency visual input^10–12^. But several studies have demonstrated that a subset of neurons in V1 can reliably respond to surface luminance (surface response)^8,13–17^. There are two different hypotheses about the generation of surface response in V1: filling-in and feedforward hypotheses. According to the filling-in hypothesis, surfaces are perceived through a neuronal mechanism that requires filling-in signals originating from responses to edge contrast. According to this view, V1 neurons with receptive fields (RF) on an object’s surface are mainly activated through horizontal connections by distant neurons whose RFs are on the object edge^18–20^. The feedforward hypothesis is that surface responses are independent of the contrast edge and directly driven by diffuse light through feedforward input^14,16,17,21,22^.

The above two hypotheses emphasize different neural circuits in V1. The feedforward hypothesis mainly relies on feedforward connections, while the filling-in hypothesis depends on long-range horizontal or feedback connections from higher-level brain areas. V1 is known to have six layers with different response characteristics^23–25^ and connection structures^26^. The input layer (layer 4 C) only has local recurrent connections and feedforward projections from the lateral geniculate nucleus (LGN)^27–29^. The output layers (layer 2/3 and layer 4B) have rich horizontal and feedback connections^30–33^. Therefore, if surface responses are generated in a way consistent with the filling-in hypothesis, we expect them to emerge in layer 2/3; on the other hand, if the feedforward hypothesis is correct, surface responses should be seen across all V1 layers.

In order to understand how the visual cortex processes edges and surfaces, it is important to investigate the transformation of the neuronal signals from the input to the output layers of the cortex. However, up to now, there has been a little physiological study of this topic. We recorded neural activity simultaneously in all layers of alert monkey V1 activated by squares of uniform luminance. We found that V1 surface responses and edge responses were distinct neural signals: both were modulated by cortical nonlocal inhibition. Furthermore, cortical inhibition switched the luminance coding strategy in V1. Luminance information was carried mainly by surface responses in the V1 input but then was encoded mainly in edge responses at the output of V1.

## Results

**Fig. 1:**
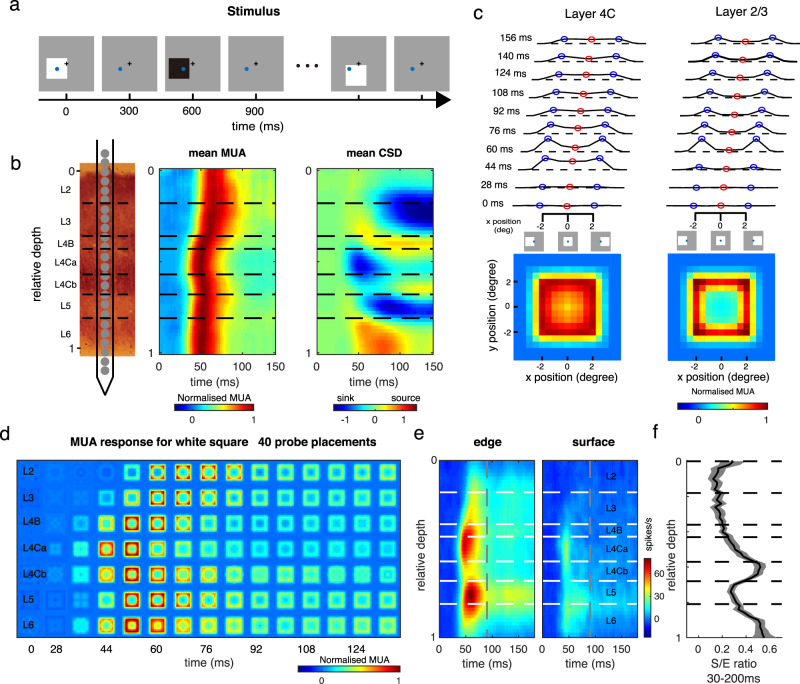
Cortical presentation of surface and edge in V1 layers. **a** Stimulus paradigm. During a trial in the fixation task, 4 × 4° black and white squares were presented, one by one, at random locations on a gray screen. Each black or white square was presented for 300 ms and followed by 300 ms blank condition. The squares had either negative contrast or positive contrast (90%) relative to the background luminance. The black plus and blue circle represent the fixation point location and a recorded site’s receptive field (RF). **b** Laminar recording and assignment. Left panel: The linear array (U-Probe, Plexon, 24 channels, interchannel spacing 100 μm) was positioned vertically through the full depth of V1. Middle and right panel: Laminar pattern of MUA and CSD averaged overall probe placements. Horizontal black dashed lines indicate the laminar boundaries in V1. **c** Dynamic of neural responses to different positions of a white square in layer 2/3 (the left panel) and layer 4 C (the right panel). response curves were plotted every 16 ms, from 28 ms to 156 ms after stimulus onset. Blue points represent the responses to the square edge. Red points represent the responses to the center of the square. Dashed lines indicate the responses to a blank stimulus. Lower panels: Spatial response patterns in layer 2/3 and layer 4 C averaged over time. **d** The plot consists of 7*17 square-like response patterns as in **c** (averaged between 40 probe placements). Each one of these patterns represents a snapshot of normalized population-averaged responses (MUA) to different positions of a white square at a particular time interval (columns: with an 8 ms time window) and at different cortical layers (rows: layers 2, 3, 4B, 4Ca, 4Cb, 5, and 6, respectively). Responses were normalized with peak response of each layer and was coded by color. **e** Laminar pattern of the neuronal response to stimulus edge (left) and surface (right). **f** Solid curve and shading represent mean ± s.e.m. of S/E ratio (ratio of surface response to edge response) smoothed by a window at 0.08 units at each cortical depth, *n* = 630. Source data are provided as a Source Data file.

The experiments utilized multi-electrode recordings in awake, behaving monkeys. Five macaque monkeys were trained for a fixation task. During each trial, a serial of black and white squares was presented, one by one, randomly at different locations around receptive fields (RFs) of the recorded sites (Fig. 1a). The size of all squares in this study was 4 by 4°, which is 5–8 times larger than the classical RF size of V1 neurons (average 0.65 ± 0.16° diameter; mean ± SD) recorded at the eccentricities of 1–5° in the present study, and fully covered the extraclassical receptive fields of the neurons (Supplementary Fig. 1). Each square was presented for 300 ms, similar to our standard fixation duration in daily life^34^. Stimulus presentation was followed by a screen presented at mean luminance (blank) for 300 ms (Fig. 1a). With a linear array (U-probe, 24 channels, 100 μm between adjacent channels), we recorded spiking activity and the Local Field Potential (LFP) simultaneously throughout the depth of V1 (Fig. 1b). For each probe placement (recording session), we assigned relative cortical depth for each of the probe’s 24 channels based on current source density (CSD) analysis^35,36^ of visually evoked LFP and the latency of stimulus-driven multiunit activity (MUA) (Fig. 1b, c). The boundaries between layers were defined by the signatures of MUA and CSD patterns and previous anatomical studies (see Method for details)^25^.

### Neural presentations for edge and surface are different in V1

To understand the neural representation of a square stimulus in V1, we estimated a recorded site’s responses to different parts of the square as a function of time, *R*(*x*,*y*,*t*) (Fig. 1c, d for a white square as the visual stimulus). Variables *x* and *y* are spatial locations of a square’s different parts relative to its center. The variable *t* is the time after stimulus onset (see details in the Method section). *R*(*x*,*y*,*t*) at each time point (Fig. 1d) tells us how a recorded site responds to different parts of a square. For instance, the response to the square’s edge, *R*_*edge*_(*t*), is *R*(*x*,*y*,*t*) with |*x* | = 2 or |*y* | = 2 (blue circles in Fig. 1c illustrate conditions with |*x* | = 2); the response to the surface, *R*_*surface*_(*t*), is *R(x,y,t)* with |*x* | ≤ 0.5 or |*y* | ≤ 0.5 (red circles in Fig. 1c illustrate conditions with |*x* | = 0)). The temporal dynamics of the two-dimensional response patterns, *R*(*x*,*y*,*t*), across V1 layers demonstrate that, shortly after stimulus onset, a square-like activity pattern first appeared in layer 4 C and layer 6. Such activity patterns gradually showed up in layer 4B, layer 2/3, and layer 5 (Fig. 1d; see Supplementary Fig. 2 for a black square as the visual stimulus).

We observed a clear difference in the pattern of square-evoked responses in the input layer (layer 4 C) from that observed in the output layers (layer 2/3). While neural activation in the input layer was comparable for surface and edge (left panel in Fig. 1c for *R*_*average*_(*x*,*y*) of layer 4 C), the response pattern in output layers looked different. The visual-evoked activity was much larger at edge regions and there was a hole in the activity map at the center of the square (right panel in Fig. 1c for *R*_*average*_(*x*,*y*) of layer 2/3). The spatial pattern in the output layers was different from our perception of a solid surface. Responses to the edge were activated across all cortical depths, but responses to the surface were activated only in the input layer and substantially decreased in the output layers (Fig. 1e). Sites with significant responses to the uniform surface in L2/3 (50%, based on signal to noise ratio > 3; see Methods) were much less than in L4C (82%). To capture the difference between edge response and surface response across V1 layers, we calculated the ratio between surface and edge response (S/E ratio) in MUA, i.e., spike activity (Fig. 1f). The S/E ratio in L4C is significantly higher than that in L2/3 (0.35 for L4C, 0.12 for L2/3, *p* < 0.01).

Interestingly, there was a large diversity of V1 responses to surface luminance. Some neurons showed transient response to surface luminance, while some neurons showed a late increment of surface response (red circle in Fig. 1c). Intuitively, this phenomenon might appear to be the result of neuronal filling-in in V1, but we found that this is not the case.

### ‘Filling-in’ of surface response starts in the V1 input layer

**Fig. 2:**
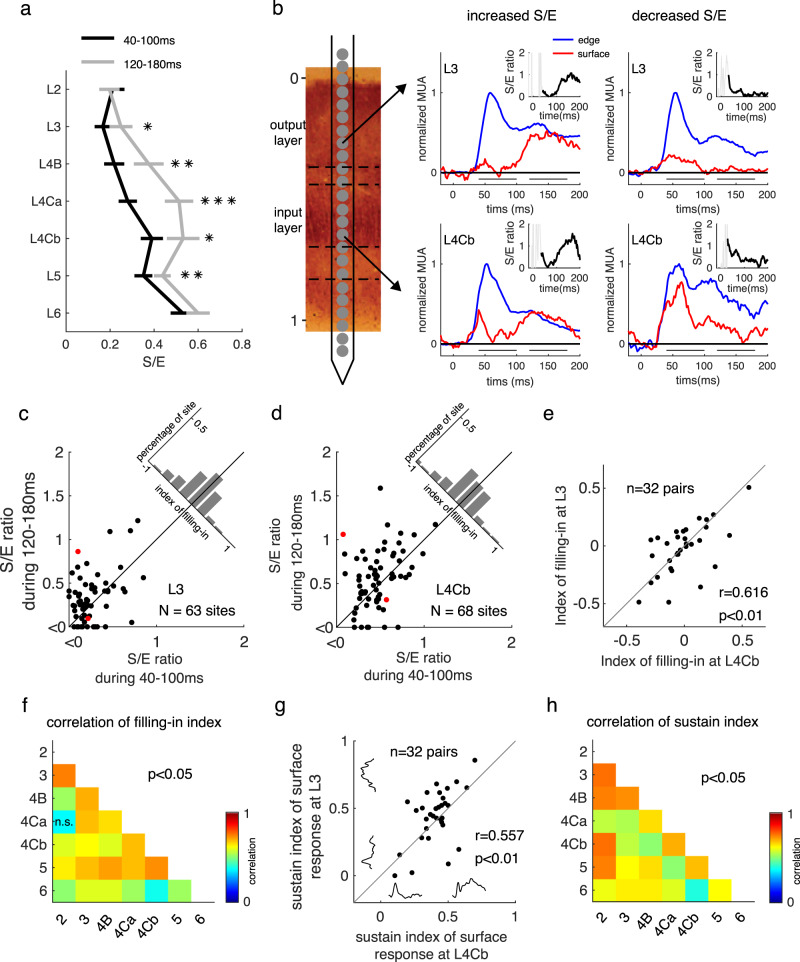
Dynamics of surface responses across V1 layers and their relationship. **a** Ratio between surface response and edge response (S/E) at 40–100 ms and at 120–180 ms after stimulus onset in different layers, averaged across sites from the 18 probe placements with increased S/E in output layer 3 or 4B. S/E ratios are presented as mean ± s.e.m. *p < 0.05, **p < 0.01, ***p < 0.001, one-sided paired *t*-test, *n* = 29, 32, 20, 34, 27, 41, 42 sites in layers from L2 to L6, respectively. **b** Two examples of dynamic MUA responses to square surface (red lines) and edge (blue lines) at output and input layer are simultaneously recorded in the same probe placement. The first example (mid-column) shows that the S/E ratio in both input and output layer increased over time in a probe placement. The other example shows that the S/E ratio in both input and output layer (right column) decreased over time in another probe placement. The early response period and the late response period were marked as solid lines in each plot. Dark lines in insets represent the dynamics of the S/E ratio from 40–200 ms. **c**, **d** Scatter plot for S/E ratio at the early time versus late time for all recorded sites at the output layer and the input layer. Red dots in figure c and d is calculated from data shown in panel **b**. **e** Correlation of filling-in strength between sites at L4Cb (*x*-axis) and sites at L3 (*y*-axis) simultaneously recorded from the same probe placement. *n* = 32 pairs, Pearson’s *r* = 0.616, *p* = 0.0002. **f** Pearson’s correlation of filling-in strength among simultaneously recorded sites at different V1 layers. *p* < 0.05 except the one marked with n.s. for *p* = 0.091. **g** Correlation of surface response sustainability between sites at L4Cb (x values) and sites at L3 (y values) simultaneously recorded from the same probe placement. *n* = 32 pairs, Pearson’s *r* = 0.557, *p* = 0.0009. **h** Pearson’s correlation of surface response sustainability among simultaneously recorded sites at different V1 layers. All *p*-values are <0.05. Source data are provided as a Source Data file.

The delayed increment in surface response has been considered a filling-in signal induced by the neural response to the edge contrast. According to the hypothesis of neuronal filling-in, facilitation of the S/E ratio should be observed in the V1 output layer but not the input layer where horizontal and feedback connections were scarce. To quantify the increment precisely, we used the S/E ratio as the indicator for neural filling-in for the square surface. To our surprise, compared to the S/E ratio at early times (40–100 ms), we observed an increment of the S/E ratio at the late time (120–180 ms) after stimulus onset in both input and output layers (Fig. 2). Furthermore, the change in S/E ratio seemed consistent between output layers and input layers: a probe placement with increased S/E ratio over time in layer 3 also tended to have an increased S/E ratio in layer 4Cb (Fig. 2a, b).

To test whether the filling-in at the output layer is inherited from that at the input layer, we quantified the filling-in strength as the change of S/E ratio through time (120–180 ms versus 40–100 ms after stimulus onset) and compared data simultaneously recorded from input layers and output layers (Fig. 2c, d). The filling-in strength was positively correlated between layers 4Cb and layer 3 (*r* = 0.62, *p* < 0.01), and there was no significant difference between input layers and output layers (Fig. 2e and Supplementary Fig. 3). The correlation was also significant among all pairs of V1 layers (Fig. 2f).

Late increased S/E ratios can be caused by a decrease/adaptation of edge responses or increase/persistence of surface responses. To understand the main cause for the change of the S/E ratio, we also correlated the strength of filling-in to the sustain index (SI) (see the definition for sustain index in Method) for both surface responses and edge responses. We found that filling-in strength was highly correlated with the dynamics of surface response in all layers (Supplementary Fig. 4). The further facilitation of surface response led to an increased S/E ratio in both input layers and output layers. Consistent with the change of S/E ratio, the sustainability of surface response also was correlated among V1 layers (Fig. 2g, h).

Taken together, the appearance of filling-in-like surface responses in the V1 input layer and the significant correlation of filling-in between the input layer and output layer indicated that the sustained surface response in V1 output layer is likely inherited from that at the input layer, rather than being induced by the edge response through horizontal connections in the output layer.

### Surface luminance is coded differently in V1 input and output layers

**Fig. 3:**
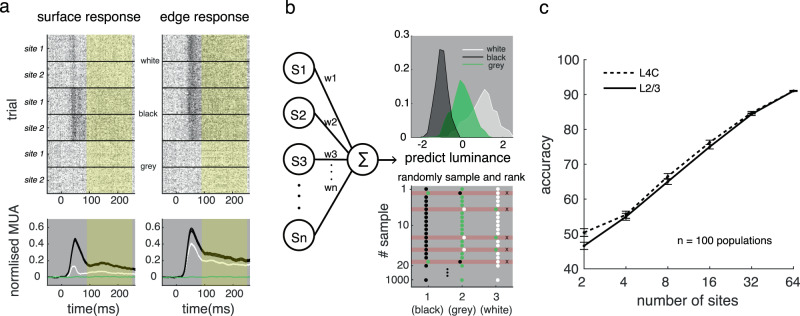
Schema of the population luminance decoding model. **a** MUA dynamic response to square edge and surface in V1. Upper: Spike rasters of two recorded sites from L4C evoked by square surface and square edge. From top to bottom: responses to white square, responses to black square, and responses to gray background. Lower: Mean dynamic response (shadings represent ±s.e.m.) in L4C to the surface and edge of black square (black), to those of white square (white), and to the gray background (green). The yellow area indicates the period for signal used in following decoding processing. **b** The decoder for square luminances. Left: a linear decoder to predict the stimulus’s luminance with a weighted summation of population MUA response. Upper right: the distribution of predicted luminances for white, gray, and black. Lower right: the accuracy of the decoder is measured by bootstrap method. Colored points (black, green and white) are randomly sampled from the three distributions shown in upper right panel, and red shadows marks wrong predicted samples. **c** Decoding accuracy for luminances in L4C (dashed line) and L2/3 (solid line) increased with the number of sites included. Error bars represent ±s.e.m. The mean and s.e.m of the decoding accuracy at a given number of sites (x values) was calculated based on decoding accuracies of 100 neural populations. Each of the neural population consists of sites at the given number (x values) randomly sampled from all sites in L2/3 or L4C. Source data are provided as a Source Data file.

A critical question is how surface luminance information is coded in neuronal responses at different times and in different layers. This question cannot be answered with the average neural response; we had to analyze the ability of the neural population to interpret the square luminance. Referring to previous studies of decoding^7,37,38^, we trained a linear decoder to predict the stimulus’s luminance by a weighted summation of population MUA response cumulated in the whole trial (Fig. 3a; see details in Methods). The data were aggregated from different probe placements and were normalized with the maximum response of each site. After training the decoder, we obtained the distributions of predicted luminances for the three conditions (white, black, and gray/background luminance equal to 1, −1, and 0, respectively). Then we evaluated the decoder’s performance for luminance discrimination with the rank order of predicted luminance. (Fig. 3b, see Methods for details). As expected, the decoder’s performance increased with the number of sites included in both input and output layers (Fig. 3c).

**Fig. 4:**
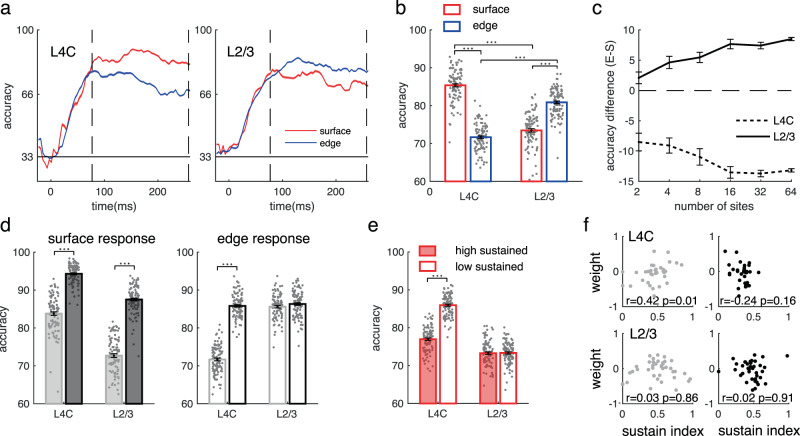
Decoding accuracy for square luminances by responses to edge or surface in V1. **a** Temproal development of decoding accuracy for luminance (shadings represent ±s.e.m.) based on surface responses (red lines) or edge responses (blue lines) of neural populations (*n* = 100) in L4C or L2/3. Each population include 32 sites randomly sampled from all sites in L4C or L2/3. The accuracy during the sustained response time (80–250 ms, between two dashed vertical lines) were averaged for the following comparison (**b**–**e**). **b** Performance of luminance decoders using surface responses (red bars) and edge responses (blue bars) in L4C and L2/3. The number of sites for each decoder was kept constant at 32. ***p < 0.001, two-sided paired *t*-test (between surface and edge) and independent *t*-test (between L4C and L2/3). Bars represent mean accuracy across populations (±s.e.m.), with individual data (*n* = 100) superimposed. The definitions for bars in **d** and **e** are the same as that for **b**. **c** Accuracy differences for decoding luminances between surface response and edge response in L4C and L2/3 are consistent regardless of the number of sites included in the decoder. Error bars represent s.e.m. across 100 populations. **d** Decoding performance for luminance recognition of white (white bars) or black (black bars) by surface responses (left) or edge responses (right) at input or output layers. ***p < 0.001, two-sided paired *t*-test, *n* = 100 populations. **e** Decoding performance of surface data with high sustainability (filled bars) or low sustainability (open bars). ***p < 0.001, two-sided independent *t*-test, *n* = 100 populations. **f** The correlation between sustainability of surface response (*x*-axis) and decoding weight (*y*-axis). The relationship between decoding weights and sustain index of response were shown in the subplots with light dots (left) for white conditions and in the subplots with dark dots (right) for black conditions. The upper panels show data from the input layer, and the lower panels show data from the output layer. Pearson correlation coefficient (*r*) were calculated from the sites with decoding weight not equal to 0 (*n* = 35 for L4C; *n* = 41 for L2/3). Source data are provided as a Source Data file.

Next, to understand precisely how the two types of neuronal responses carry surface luminance in different layers, we trained decoders based on responses to stimulus edge or surface separately. We binned the neuronal response at a resolution of 50 ms and estimated the decoder performance over time. The performance of the decoders increased from chance level after stimulus appearance and remained relatively stable over the time of the trial (Fig. 4a).

The accuracy for coding luminance is not always consistent with the neural response strength. Although the surface response is weaker than the edge response in the input layer, the surface response has better accuracy for coding luminance information. However, in the output layers, the best readout of surface luminance goes to the edge response (see Fig. 4b for averaged decoder’s accuracy during the period from 80 to 250 ms). The laminar difference of coding strategies for luminance information by surface and edge responses remains unchanged for decoders with different numbers of sites included (Fig. 4c). There was an asymmetry in coding accuracy for a black surface versus a white surface. In the input layer, both surface and edge responses recognize black better than white. However, in the V1 output layer, only the surface response shows a large black dominance for luminance recognition (Fig. 4d).

Given that there is a variety of filling-in strengths for the neural population (Fig. 2c), another critical question is whether or not sites with the more sustained surface response (with stronger filling-in strength) carry more luminance information than other sites (that is, do they have a larger weight in the decoder)? Separating surface response as a low-sustained set and a high-sustained set, we found that the population with high sustainability performed similarly to the low-sustained population (Fig. 4e; accuracy is 73.28 versus 73.34 on average in L2/3, *p* = 0.87). Consistently, we found that there was no significant correlation between the decoding contribution of a site and the sustainability of its surface response in the V1 output layer (Fig. 4f). Thus, continuous reaction to the object surface did not enhance the population recognition of the surface luminance in V1.

The above decoding analysis is conditioned upon the assumption that V1 downstream neural populations treat inputs driven by surface and edges differently and V1 populations downstream can tell whether their inputs are from stimulus surface or edges. To confirm this idea, we also built a decoder that treated surface response the same as edge response, and found that luminance reading accuracy was significantly reduced (Supplementary Fig. 5). This suggests that V1 has distinct surface and edge responses that contain different visual information; and in order to readout luminance information correctly from neural activity, a downstream area needs to distinguish responses driven by surface and edge. To demonstrate that this is feasible, we further built a ‘multiplexed’ decoder to identify stimulus locations as well as stimulus luminances (Supplementary Fig. 6). We found that a V1 population contains information for both location and luminance information for a stimulus. Interestingly, the reading of location information is significantly earlier than that of luminance information, which suggests that the downstream area might prioritise the reading of location information to guide the reading of luminance^4,39^ (see details in supplementary method and Supplementary Fig. 6).

Results in this section revealed that, in V1 output layers, the coding for luminance information starts switching to edge responses from surface responses. The decrement of coding accuracy for luminance information by surface response is generally true, regardless of whether or not the surface response has a late increment. Our next goal was to find out the neural mechanism that leads to the decreased surface response (Fig. 1f, g) and the switch of coding strategies for luminance from the V1 input layer to the output layer (Fig. 4b).

### Responses to edge and surface are both suppressed by the surface in V1 output layers

To understand the underlying neural mechanisms of surface and edge processing, we dissected the excitation and inhibition components around the operation point from the neuronal activity using reverse correlation (see details in Methods). The method linearized the neuronal activity associated with surface and edge stimulus^25,40^. The reverse correlation experiment was similar to the flashed experiment with two different settings: (1) each square was presented only for 20 ms; (2) to measure the interaction between surface luminance and edge response in V1, we also added stimuli with edge components only (a 4 × 4° square frame).

**Fig. 5:**
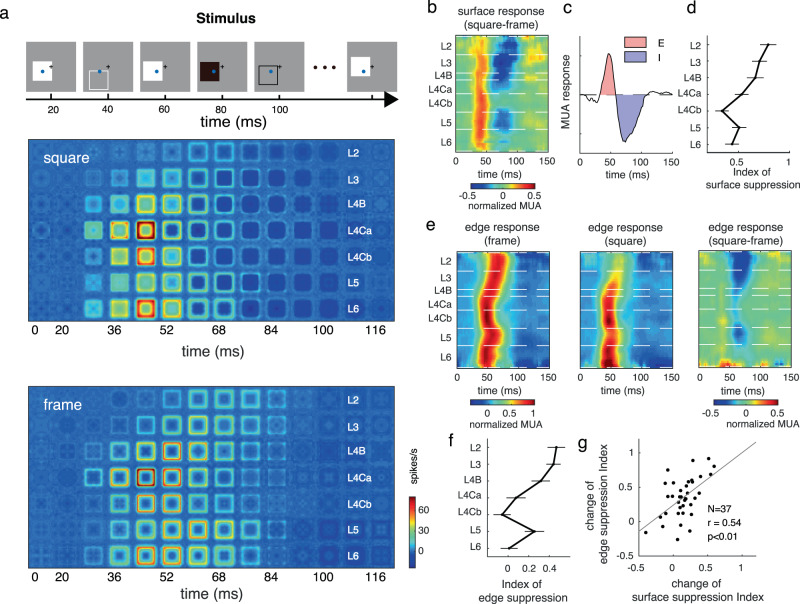
Responses to surface and edge are both suppressed by surface luminance. **a** Measuring the laminar pattern of dynamic responses to squares and frames. During a trial of fixation tasks, 4 × 4° squares or frames were rapidly and randomly presented around the recorded sites’ receptive field on a uniform gray screen. All stimuli were represented for 20 ms, one by one, at random locations. The laminar pattern shows MUA response to white square (left) and white frame (right) in different V1 layers, averaged between 11 probe placements. **b** Laminar pattern of MUA response to the square surface. **c** Dynamic MUA response to the square surface. The surface response has typical early positive (shaded red) and late negative part (shaded blue). The excitatory component is defined as positive response strength, and the inhibited component is defined as negative response strength. **d** Laminar distribution of surface suppression. Surface suppression is defined as inhibited component divided by the sum of inhibited component and excitatory component. Error bars show s.e.m. across sites. *n* = 14, 23, 11, 20, 20, 19, 22 sites in cortical layers from L2 to L6, respectively. **e** Laminar pattern of MUA responses to the frame edge (left), square edge (middle), and responses difference between square edge and frame edge (right). **f** Laminar distribution of edge suppression. Edge suppression is defined as frame edge response subtract from square edge response then divided by frame edge response. Error bars show s.e.m. across sites. *n* = 14, 23, 11, 20, 20, 19, 22 sites in cortical layers from L2 to L6, respectively. **g** Correlation between surface suppression change and edge suppression change at the output layer. *n* = 37 pairs, Pearson’s *r* = 0.54, *p* = 0.0005. Source data are provided as a Source Data file.

Similar to the results from the flashed experiment, in the reverse correlation experiment, the response to the square surface was relatively strong in the input layers and decreased in the output layers (Fig. 5a). Neurons responding to the square surface showed strong negative responses following the initial positive responses (Fig. 5b, c). The suppression index (defined as the ratio of the negative component to the sum of the negative and positive components) in the output layer was notably larger than that in the input layer (Fig. 5d). As a control, neurons responding to the center of the frame did not have any significant response (Fig. 5a). This finding suggests that the weaker surface response in L2/3 were due to cortical inhibition.

Next, we compared the responses to edges with (square edge) or without (frame edge) a surface. We observed different interlaminar transmission for neural responses to the square edge and frame edge (compare Fig. 5a upper and lower). The response evoked by the square edge was significantly weaker than that produced by the frame edge in the output layer (Fig. 5e, f; *p* < 0.01, two-sided paired *t*-test), but responses evoked by the two types of edges were not significantly different in the input layer (Fig. 5f; *p* = 0.8, two-sided paired *t*-test). That result indicates a suppressive effect on the square edge (Fig. 5e). We measured the intensity of suppression on the edge response with an index defined as the difference between the response strength of the frame edge and the response strength of the square edge. Suppression of edge response showed a similar laminar profile as the inhibition of the surface response (Fig. 5f). Furthermore, from the input layer to the output layer, increased surface suppression was significantly correlated with the increased edge suppression (Fig. 5g, *r* = 0.54, *p* < 0.01). This result suggests that a general cortical inhibition might play an essential role in modulating surface and edge response.

### Cortical inhibition evoked by the surface is nonlocal and luminance-dependent

**Fig. 6:**
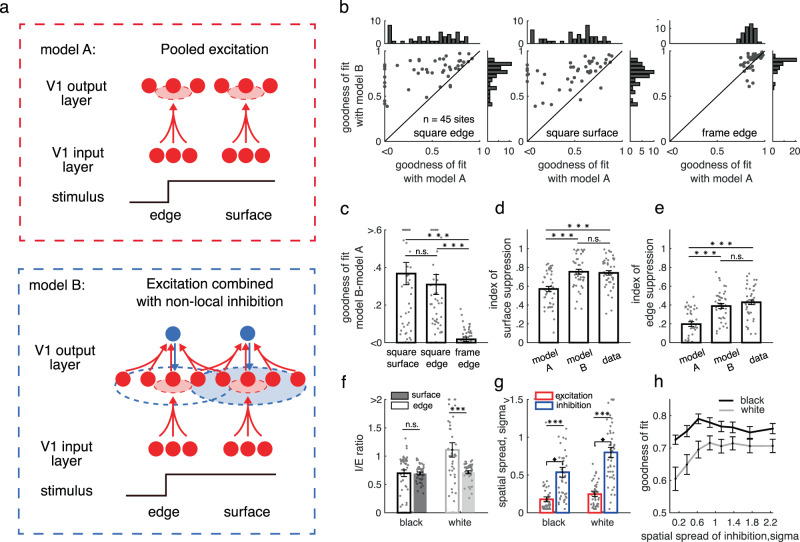
Cortical mechanism achieving processing of object surface and edge. **a** Illustration of two possible models for the generation of surface responses in the output layer. Model A (upper panel), neuronal responses in the output layer are generated by pooling excitatory projections from the V1 input layer. Model B (lower panel), neuronal responses in the output layer are generated by combining the excitations and nonlocal inhibitions in the output layer. **b** Performance comparison for explaining the neuronal response in the output layer between model A (*x*-axis) and model B (*y*-axis). *n* = 45 sites from output layers 2, 3 and 4B (same for **c**–**h**). **c** Performance improvement from model A to model B. ***p < 0.001; n.s.: *p* = 0.471, two-sided paired *t*-test. Bars (in **c**–**g**) present mean of corresponding values across sites (±s.e.m.), with individual data superimposed (*n* = 45). **d**, **e** Predicted surface suppression (**d**) and edge suppression (**e**) from the two models. ***p < 0.001; n.s.: *p* = 0.2806 (in d) and *p* = 0.0973 (in **e**); two-sided paired *t*-test. **f** Nonlocal inhibition strengths for edge (open bars) and surface responses (filled bars) to black (black bars) and white square (white bars). Surface response to a white surface has the strongest inhibition. ***p < 0.001; n.s.: *p* = 0.9412; two-sided paired *t*-test. **g** Spatial extension for pooled excitation (red bars) and nonlocal inhibition (blue bars) for the black and white signal. ***p < 0.001, two-sided paired *t*-test. **h** Model performance with constraints for different spatial ranges of inhibition. Error bars show s.e.m. across sites. Source data are provided as a Source Data file.

To test the hypothesis that a common cortical inhibition might modulate the laminar transmission of both edge and surface responses at the V1 output layer, we compared two computational models for their predictions of dynamic responses. Model A (the upper panel in Fig. 6a) generates neural responses in the V1 output layer only by pooling excitation from the V1 input layer. Model B (the lower panel in Fig. 6a) integrates excitations from the input layer and gets modulated by intralaminar inhibition. In both models, the integration of excitation from the input layer was modeled as a spatial-temporal convolution of neural responses in the input layer. We assumed that the linear filter is spatial-temporal separable. The spatial feature was modeled as Gaussian kernel, and the temporal feature was modeled as a log-transformed gaussian kernel (see Methods for details). We further assumed that the gain of the linear filter depends on stimulus positions (surface vs. edge of a stimulus). For model B, intralaminar inhibition was modeled in a way similar to excitatory processes. We optimized model parameters by minimizing the MSE between predicted and real response dynamics of V1 output layer (see Methods for details of the two models).

As we expected, model A with excitation alone did not predict response in the output layer (Fig. 6b; mean goodness of fit is 0.4 for square and 0.4 square edge). Model B (mean goodness of fit is 0.78 for square surface and 0.75 for square edge) was much better than model A for predicting dynamic responses in the output layer, especially for responses to square surface and edge (Fig. 6b, c). The intralaminar inhibition induced in model B also generated comparable suppressions on surface response and edge response as observed in data (Fig. 6d, e).

We found that cortical inhibition had a nonlocal effect. The spatial spread, estimated with the sigma of the gaussian function, of the cortical inhibition was about two times larger than the spread of pooling excitation, on average (Fig. 6g). We further estimated the fitting performance of the model as a function of the range of inhibition (Fig. 6h). When the range of cortical inhibition was about 0.6–0.8 degree in sigma, the predicted V1 response was significantly closer to the real data. These results suggest that the neural mechanism for surface and edge response in V1 output layers is an interaction of local feedforward excitation and nonlocal inhibition.

Our results also suggest an asymmetry of black and white in nonlocal inhibition (Fig. 6f). The inhibitory strength (I/E ratio, defined as the ratio between inhibition and pooling excitation in Fig. 6f) for white stimuli (I/E ratio: 1.1 ± 0.13) was significantly larger (*p* < 0.01, two-sided paired *t*-test) than for black stimuli (I/E ratio: 0.74 ± 0.06). The spatial spread of the cortical inhibition elicited by a white surface also is wider (0.80 ± 0.06 for white surface and 0.53 ± 0.05 for black surface, *p* = 0.016, two-sided paired *t*-test Fig. 6f). The distinct strength and spatial scale of cortical inhibition may explain enhanced black dominance of surface response compared with edge response (Supplementary Fig. 7).

### Cortical mechanisms for processing luminance information of object surface

Finally, we examined how the dissected cortical excitation and inhibition influence the change of coding strategies for surface luminance from the V1 input layer to the output layers. We applied model B for fitting the trial averaged dynamic responses to a square surface and edge presented for 300 ms (dataset shown in Figs. 1, 2) in the V1 output layer. Model B could well reconstruct responses in 300 ms flashed square experiment (goodness of fit is 0.78 on average). The model captured the altered latency and sustainability of neuronal response in the output layer compared to the input layer (Supplementary Figs. 8, 9). Next, we generated single-trial responses based on model parameters and noise estimated from each recorded site at the output layer (see details in Methods); and evaluated the coding ability for luminance information in model B (similar to what we did for experimental data shown in Fig. 4).

**Fig. 7:**
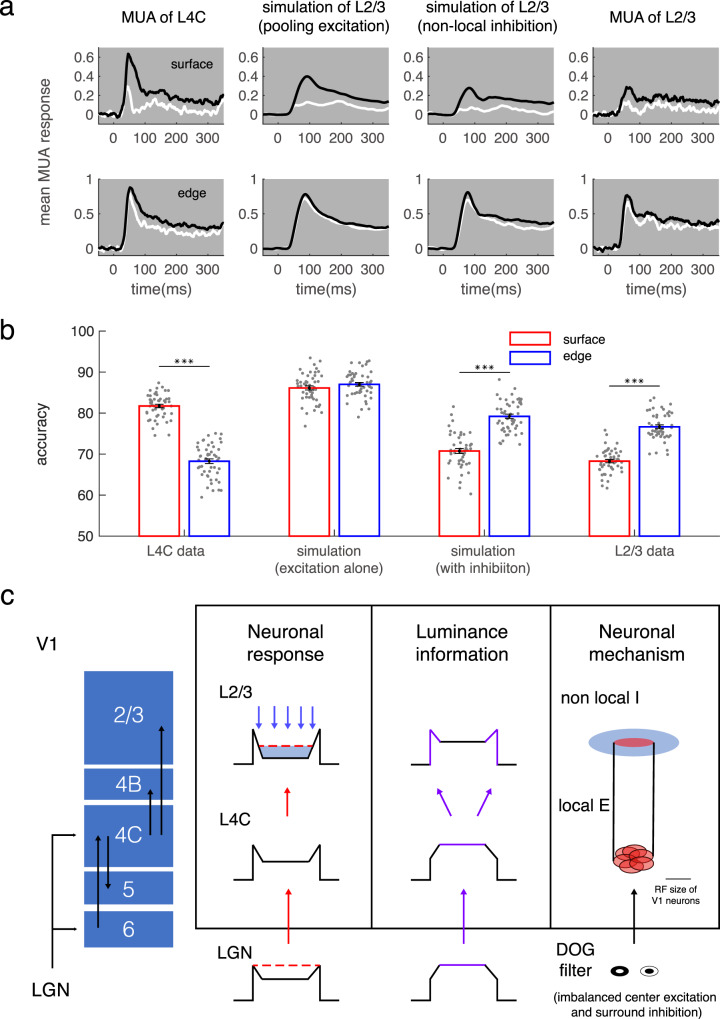
The cortical mechanisms for switching coding strategy of surface luminance. **a** Population-averaged dynamic response (shading for ±s.e.m.) to surface (upper row) and edge (lower row) of black and white squares in L4C (first column), in model A for L2/3 (second column), in model B for L2/3 (third column), and in L2/3 (fourth column). **b** Decoding performance for surface luminance under the 4 conditions in **a**. The number of sites for each decoder was kept constant at 32. ***p < 0.001, two-sided paired *t*-test. Bars show mean accuracy across populations (±s.e.m., *n* = 50), with individual data superimposed. **c** Schematic illustration for changes of neuronal responses and coding strategy for luminance information. Neurons in L2/3 integrate pooled projection, illustrated by red arrows, from L4C in a small cortical range and are modulated by inhibition, shown by blue arrows, in a more comprehensive cortical range. The surface luminance information was illustrated by purple color in the ‘luminance information’ column. Source data are provided as a Source Data file.

To separate the respective effects of pooling excitation and inhibition, we generated two groups of data sets: one simulated with pooling excitation alone, another simulated with the combined excitation and inhibition (Fig. 7a, the 2nd and 3rd panel). We found that these two cortical processes play different roles in signal transmission and can redistribute surface luminance information in V1 (Fig. 7b). The pooling of excited inputs improves the luminance recognition ability based on edge response (Fig. 7b, accuracy is 68 versus 87 on average, *p* < 0.01). Inversely, the following intralaminar inhibition reduced the luminance recognition ability based on surface response (Fig. 7b, accuracy is 86 versus 70 on average, *p* < 0.01). In summary, a center-surround antagonistic structure constructed by local excitation and nonlocal inhibition makes the edge response dominate in surface luminance interpretation.

## Discussion

Our study provides a complete picture of dynamic laminar processing for surface luminance and edge contrast in macaque V1. The distinct laminar patterns of surface response and edge response enable us to reveal neural mechanisms for laminar processing of object surfaces in V1. We found that a local feedforward mechanism from the input layer and a nonlocal inhibitory mechanism in the output layer interacted to modify V1’s representation of surface luminance. As summarized in Fig. 7c, feedforward excitation drives both surface and edge responses and mainly enhances the coding accuracy of edge response for luminance information. Cortical inhibition suppresses both edge and surface responses and functionally redistributes luminance information from the input layer to the output layer in V1. We conclude that these two cortical processes, taken together, integrate surface luminance information into the edge response and result in the edge-based efficient coding of surface luminance in V1.

Previous studies offered different hypotheses for the generation of the response to surface luminance in V1. The filling-in hypothesis was supported by findings of slow latencies for surface responses, late increments of surface responses^18,20^, and late perception-related surface responses^13^ in V1. Alternatively, the feedforward hypothesis was supported by findings of independent luminance response in V1^8,16^ and the significant responses to low-spatial frequency luminance without edge contrast^22^. In the present work, we found a diversity of V1 responses to surface luminance. Some neurons, located in both the input and output layers of V1, responded significantly to a uniform surface earlier than to an edge. But other neurons had a delayed surface response, more frequently in the output layer. These phenomena, seen in the past as conflicts, can be well explained by unified neural mechanisms.

V1 neurons show changes of sensitivities to surface luminance across layers, and neurons in the input layer strongly respond to surface luminance. Based on the lack of horizontal connections in the input layer, response to the surface is likely driven by feedforward excitation from the pre-cortex. Studies have reported that pre-cortical neurons have imbalanced integrated sensitivity of center excitation and surround suppression^41,42^, as well as black/white asymmetry;^43^ and they can respond well to uniform luminance and offer sustained driving force to V1 input layers^8,44,45^. The late increased surface response, thought to be a filled-in signal induced from object edge^18^, is observed in both input and output layers here. Observations in the present work indicate that the sustained surface response in V1 is mainly inherited from feedforward input, and not induced by edge responses as a product of filling-in. The robust reaction to surface luminance in the V1 input layer is suppressed due to cortical inhibition. Inhibition received at the beginning of the response can delay the response latency and could make the later enhancement appear more pronounced. The combination of feedforward input and cortical inhibition can cause the laminar difference of surface response sensitivity and the diverse, dynamic characteristic of surface response.

One of our main findings is that nonlocal cortical inhibition modulates both surface and edge response in the V1 output layer. Such cortical inhibition is mainly induced by the object surface but not by the edge (Fig. 6). Our measurements are consistent with previous findings on the suppressive effect of transient luminance change in V1^12,46,47^. One remaining question is what circuitry generates the cortical inhibition evoked by surface luminance. Intracellular recording in the primary visual cortex of awake tree shrew found that a transient change of luminance increases the inhibitory conductance of layer 2/3 neurons without a change in the excitatory conductance^12^. Consistent with the morphologic evidence that inhibitory neurons in layer 4 of V1 can supply feedforward inhibition to layer 2/3 neurons^48^, the work of Tucker and Fitzpatrick (2006)^12^ suggests that luminance-evoked inhibition is triggered by feedforward inhibitory projections. The significant correlation between neural activity in the input layer and the cortical inhibition in layer 2/3 (*r* = 0.52, *p* < 0.01) suggest that feedforward inhibition is a possible source. However, this significant interlaminar correlation also supports the scenario in our model that V1 input layer directly drives excitation and indirectly drives inhibition through nonlocal integration in output layers. Our study suggests that the spatial range of cortical inhibition is much larger than the V1 receptive field size and the spatial scale of feedforward projections^49^. More importantly, the inhibition scale is comparable to the extraclassical receptive field size of V1 neurons^50^, suggesting that long-range horizontal and feedback mechanisms in V1 output layers might engage in the integration of edge and surface signals^51–53^. The inhibition in our present model is generated by pooling a range of neural activity in cortical space without distinguishing edge responses or surface responses. A further study on inhibition as a function of square sizes is worth doing in order to differentiate the inhibitory modulations from stimulus surface and edges.

Consistent with previous studies^47,54^, we also found that cortical inhibition elicited by a white surface is not only stronger than that elicited by a black surface but also has a wider spatial range. The greater inhibition driven by a white surface is consistent with the enhanced black dominance of surface responses in the V1 output layers. This suggests that, besides pre-cortical mechanisms^43,55,56^, cortical inhibition might also contribute to V1 black dominance^17,40,47,57^. However, our current study was neither designed nor equipped to explore the cognitive consequences of luminance-dependent inhibition or black/white asymmetry. Further study of complex stimuli with different shapes^58^ and luminance would be worth doing.

Consistent with the textbooks, we confirmed the idea that edge responses carry information about surface luminance. What is astonishing is that the advantage of edge-based luminance encoding emerges in V1 output layers. Although the center-surround receptive field structure of neurons at the pre-cortical stage—retina and LGN—can enhance the strength advantage of the edge response, it is not enough to cause the domination of edge response in the expression of surface luminance. Here, we found that luminance information is redistributed from surface response to edge response within V1 cortex. A considerable number of studies on the primary visual cortex of human^8,59–62^, cat^63,64^, and monkey^7,13,65–67^ reported that there are retinotopic signals in response to the perception of surface luminance. How surface luminance perception is computed from the cortical population response is still unclear. There are several theoretical hypotheses^6,68,69^, but they lack direct neuronal evidence. Using a decoding approach, we demonstrated the contributions of surface and edge response in surface luminance coding: luminance information was mainly coded by surface responses in the input layer but was better coded by edge responses in V1 output layers. Also, we found that the influence of surface response on luminance recognition did not increase with time (Fig. 4a), indicating that functional ‘filling-in’ in V1 is not used to represent surface luminance.

Our results suggested that cortical inhibition causes the redistribution of luminance information to the neurons in layers 2/3 that respond to edges. Nonlocal inhibition and feedforward excitation construct a center-surround antagonistic structure. This structure reduces the redundant luminance information retinotopically carried by the surface response and integrates the information into the edge response. The center-surround antagonism illustrated in our work is consistent with the optimal decoder modeled in the V1 superficial layers by Chen, Geisler et al.^70^. The advantage of their proposed decoder is that it removes the spatial correlation of group responses. Since the data used in our decoder were not recorded simultaneously, they were not spatially correlated. Therefore, we could not estimate the improvement of luminance coding efficiency by the nonlocal inhibition alone. More work should be done by simultaneously recording neuronal responses across a large spatial scale to answer the question.

What effect do surface responses have on perception or cognition? Our study shows that surface responses are not well correlated to edge responses (Supplementary Fig. 4), and luminance information carried by responses to surface and edge are not the same (Supplementary Fig. 5). These results support the idea that the surface responses provide a distinct driving force and visual information to downstream neurons for visual cognition^58,71^. Recent V4 studies^58,71^ have shown that edge and interior surface information are indeed maintained in parallel until the midlevel visual representation. Our study focused on the neural representation of surface luminance for simple objects (squares with uniform luminance); however, surface luminance for complex objects^58^ and other information (such as textures and colors) about objects’ surfaces^71^ were not included in the study. How do surface and edge information combine/interact for object recognition in complex scenes needs further research^4^. The faster reading of location information relative to luminance information in our study (Supplementary Fig. 6) indicates that object shape might be used as the initial cue in object detection^39^, even though the surface response might be faster than the edge response (Supplementary Fig. 9).

Previous literature considered the facilitation of surface response as a filling-in signal following edge responses. In the present work, we observed the filling-in signal in both input and output layers and suggested that it doesn’t propagate from the edges. Then we have to ask, how does the ‘filling-in’ like reaction pattern take place? It may trace back to LGN. Plenty of evidence from different species showed rebound (or late increment) in LGN response^72–75^. Research on mice using the silencing method confirmed that the later rebound in V1 response was initiated in LGN but not caused by feedback from higher visual areas to V1^74^. Considering this work, we thought the rebound response observed here might be driven by afferent signals from LGN. The generation of a rebound signal in LGN was thought to rely on characteristics of relay cells in LGN^76^. Since 40% of the total synapses in LGN are contributed by corticothalamic projection from V1 layer 6^77^, the thalamocortical loop may also contribute to the generation of the rebound response in LGN.

Do cortical connections further facilitate surface responses in the output layers? Recurrent networks across different layers in V1 may amplify feedforward signals through neural oscillation there^78^. We noticed that there was a strong correlation between surface response enhancement in layer 3 and layer 5. However, our present model without this possible recurrent facilitation can already predict surface response in layers 2 and 3. Furthermore, our data and model results indicated that the main cortical effect on surface processing was the initial inhibition shortly after stimulus onset, as mentioned above, and its later facilitation was not as significant as that in the edge response. Therefore, we speculated that instead of the surface response, edge response in the output layer received functionally significant cortical facilitation.

## Methods

### Preparation and behavior task

All experimental procedures were conducted in compliance with the National Institutes of Health *Guide for the care and use of laboratory animals* and were approved by the Institutional Animal Care and Use Committee of Beijing Normal University. Five male adult rhesus monkeys were used in this research. Monkeys were implanted with a titanium head post and a recording chamber placed over the primary visual cortex under general anesthesia surgery as described previously. The animals were trained to fixate within a window of 2 degree centered on a 0.1 degree fixation point (FP) displayed in the center of the screen. We recorded eye movements using an infrared tracking system (ISCAN). A trial began when a monkey began fixation. A trial began with the FP flashed on the gray background, and the monkeys started the trial when they keep fixating on the FP. After 200 ms of fixation, the stimulus was displayed for around 3–4 s, followed by a blank interval of 300 ms. After FP disappeared, the animal received a drop of water as a reward. A trial was aborted if the animals fail to maintain fixation during stimulus presentation.

### Electrophysiological recording

We used a linear electrode array (U-probe, Plexon; 24 recording channels spaced 100 um apart, each 15 um in diameter) to record neuronal activity simultaneously from different cortical depths of the primary visual cortex. The linear array was inserted into the cortex on each day of experiments under the control of a microelectrode drive (NAN Instruments), and its depth was adjusted to extend through all V1 layers. Electrical signals from electrodes were amplified and digitized with a multichannel recording system (Blackrock Microsystems). The local field potential is defined as the low-pass-filtered (300 Hz) signal, and multiunit activity (MUA) is detected by applying a voltage threshold with a signal-to-noise ratio of 5.5 on the high-pass-filtered (1000 Hz) signal.

### Visual stimulation

Visual stimuli were presented on a 22-inch CRT monitor (Dell, P1230, 1200 × 900 pixels, mean luminance 45.8 cd/m^2^, 100 Hz refresh rate; the display color look-up table was calibrated to be linearized.). The viewing distance for monkeys was 114 cm. Four types of stimuli were used.

Sparse noise and random orientation presentations were used to measure the basic features of recorded neurons and align laminar positions. RF location and orientation selectivity were captured with sparse noise and random orientation described previously^25^. Briefly, for RF mapping, we obtained a two-dimensional map of each channel with sparse noise and fitted averaged response of each map with a two-dimensional Gaussian function to estimate the center position and radius of each RF (σ of Gaussian function). RFs of neurons in this research were located within 5° of the fovea. For orientation selectivity measuring, we got orientation tuning curves with grating patterns (4° in diameter) of different orientations randomly flashed on the center of RFs. Then we fitted tuning curves with the von Mises function (Khatri and Mardia, 1977) and used the fitted tuning curves (spaced from −90° to 90°, at 1° interval) to estimate the ratio of orthogonal responses and preferred responses (O/P ratio).

Black and white squares presented for 300 milliseconds were used to measuring neural response to stimulus edge and surface and for the decoding analyses. Black and white squares sized 4 × 4° (at least six times larger than the RF of layer 4Cα for most probe placements) were presented against a gray background (luminance 45.8 cd/m^2^) for 300–500 ms and followed by 500 ms ‘blank’ (defined as uniform frames with the same luminance as the gray background). The luminance of the white and black squares was adjusted to generate the same contrast magnitude relative to the gray background (0.9). The distance of the square center relative to the RF center was randomly chosen from 0 to 4°. The spatial range of all squares allowed us to measure neural responses on the square edge, square center, and outside the square. There were 6–10 prepared square images shown on each trial based on trial length (Fig. 1a). Each trial displayed one segment until all segments were used. There were at least 50 repetitions of each square image.

Black and white squares or frames presented for 20 milliseconds were used to measure the detailed dynamics of edge and surface response and signal transmission model prediction. Square or frame images sized 4 × 4° were presented one by one against the gray background for 20 ms of duration. Images of black and white squares are the same as the above-described ones in the sustainedly presented experiment. Images of black and white frames have the same size of squares; the frame edge’s width is 0.2 degree. All of the square images, frame images, and blanks (10% of all stimuli) were randomly chosen and consisted of a sequence. Again, there were at least 50 repetitions of each stimulus.

### Laminar alignment

The detailed method of laminar alignment is described in the previous literature^25^. Briefly, we align the relative depth of each placement based on signatures of current source density (CSD)^35,36^ pattern (calculated from LFP) and MUA pattern driven by visual stimuli mentioned above. The signatures contain the depth of the earliest current sink in CSDs, the depth of polarity inversion in CSDs, and the shallowest depth of the channel exhibiting visually-driven spiking responses. These signatures are similar between responses driven by different stimuli we used in this research and are reliable in defining boundaries between layers. Segregation of layers also refers to previous anatomic studies on the changing of orientation tuning and RF locations^25^.

### Surface and edge response

Given the original MUA response for a square stimulus is a four dimension matrix: *R*(*x*,*y*,*l*,*τ*), x and y represents the square center’s spatial location relative to the RF center, l represent the luminance of the square, *τ* = 0 represent the onset time of the stimulus. We defined a site’s absolute response to the square edge as follows:

1 $${{{{Ar}}}{{\_}}{{{edge}}}}_{v}\left(l,\tau \right)=\mathop{\sum}\limits_{1.7 < \left|x\right| < 2.3}\mathop{\sum}\limits_{\left|y\right| < 0.5}R(x,y,l,\tau )$$

2 $${{{{Ar}}}{{{{{\rm{\_}}}}}}{{{edge}}}}_{h}\left(l,\tau \right)=\mathop{\sum}\limits_{\left|x\right| < 0.5}\mathop{\sum}\limits_{1.7 < \left|y\right| < 2.3}R(x,y,l,\tau )$$

Ar_edgev defined MUA response to vertical edges of the square, and Ar_edgeh defined MUA response to horizontal edges of the square. We chose the one with the larger response amplitude from Ar_edge_v_ and Ar_edge_h_ to represent edge response. We described a site’s absolute response to the square surface as:

3 $${{{Ar}}}{{\_}}{{{surface}}}\left(l,\tau \right)=\mathop{\sum}\limits_{\left|x\right| < 0.5}\mathop{\sum}\limits_{\left|y\right| < 0.5}R(x,y,l,\tau )$$

We then defined normalized edge response and surface response, respectively, as follows:

4 $${R}_{{{{edge}}}}(l,\tau )=\,\frac{{{{Ar}}}{{\_}}{{{edge}}}\left(l,\tau \right)}{{{\max }}(\,[{{{Ar}}}{{\_}}{{{edge}}}\left(l,:\right)\,,\,{{{Ar}}}{{\_}}{{{surface}}}(l,:)])}$$

5 $${R}_{{{{surface}}}}(l,\tau )=\,\frac{{{{Ar}}}{{\_}}{{{surface}}}\left(l,\tau \right)}{{{\max }}(\,[{{{Ar}}}{{\_}}{{{edge}}}\left(l,:\right)\,,\,{{{Ar}}}{{\_}}{{{surface}}}(l,:)])}$$

The relative response strength R_edge and R_surface have values from 0 to 1, which let us compare the surface and edge, black and white response across different layers regardless of each site’s absolute responses.

We used the signal/noise ratio (SNR) of the MUA response to determine whether or not a recording site had a reliable response to a square surface or edge. We defined SNR as follows:

6 $${{{SNR}}}{{\_}}{{{edge}}}=\,\frac{{{{{{\rm{var}}}}}}({R}_{{{{edge}}}}(\,{{{signal}}}{{\_}}{{{time}}})\,)}{{{{{{\rm{var}}}}}}({R}_{{{{edge}}}}(\,{{{blank}}}{{\_}}{{{time}}})\,)}$$

7 $${{{SNR}}}{{\_}}{{{surface}}}=\,\frac{{{\rm var}}({R}_{{{{surface}}}}(\,{{{signal}}}\,{{{time}}})\,)}{{{{{{\rm{var}}}}}}({R}_{{{{surface}}}}(\,{{{blank}}}\,{{{time}}})\,)}$$

Where signal_time is 0–300 ms after the stimulus onset and blank_time is 0–50 ms before the stimulus onset. And we defined a site with SNR larger than 3 to have a reliable response to the stimulus. Response sustainability is represented by the sustain index (SI). SI is determined as a ratio of the integrated response during 120–200 ms after stimulus onset to the integrated response during 40–200 ms after stimulus onset. SI close to 1 indicates a later increase/sustain for the response, and SI close to 0 indicates a strong decrease/adaptation for the response.

### Surface luminance decoding

We made a linear regression between the population response and the corresponding stimulus’s luminance to get the weight matrix of the decoder. The population response matrix is defined as X, X = (X_1_, …, X_N_)^T^, where X_i_ is a m (m is total number of trials) by 1 vector of the MUA response of neuron i averaged in a chosen time bin, and N is the number of neurons in the population. We aggregated all the data together for the decoding operation. We trained the linear regression model to fit population response to the square luminance:

8 $$L=X{{{{{\bf{W}}}}}}+w0$$

where L is a #trials by 1 vector of square luminances, W is an N by 1 vector of weights, and w0 is a #trials by 1 vector of scalar affine terms. The weight matrix (W and w0) of the decoder were optimized to minimize the mean square error (MSE) between the estimated luminance and the actual luminance, by ﻿fminunc function in Matlab.

9 $$J=\,\frac{1}{m}\mathop{\sum }\limits_{i=1}^{m}{({Y}_{i}-L)}^{2}+\frac{\lambda }{m}\left|{{{{{\bf{W}}}}}}\right|$$

Here, we used an L1 regularization term to minimize the obtained weights and prevent overfitting. We randomly separated the dataset into a training set (60%), a validation set (15%) and a test set (25%). The regularization parameter (lambda) was chosen as the one that gave the lowest MSE in the cross-validation test among 20 lambda values equally spaced (logarithmically) between 0.001 and 40 (Supplementary Fig. 10). The main results shown in this paper are based on the regularization parameter at 0.7 for all sites.

The decoder’s accuracy for luminance discrimination was calculated with ranking performance for predicted luminances. We randomly sampled a predicted luminance value from each of the three stimulus conditions (black, white and gray﻿) and ranked the three values. The smallest value was marked as black, the middle ones as gray, and the largest ones as white. If the ranking order of the sample set is consistent with the true order of stimulus luminances, the result of the luminance decoding of the sample set is correct, otherwise, it is wrong. We repeated this procedure 1000 times with a bootstrap method and calculated decoding accuracy as the ratio of correctly ordered sample sets out of the total number of sample sets. The performance of cross-validation under optimal regularization parameter is up to 86% accuracy on average. We also trained a decoder with nonlinear processing after linear regression (linear-nonlinear (LN) decoder), and found no increase in the decoder performance (Supplementary Fig. 11), so we only report the result using the linear decoder.

To test the performance of the optimal decoder as a function of time, we binned the neuronal response at a resolution of 50 ms and estimated the decoder performance over time. We simultaneously trained the decoder separately on each 50 ms time bin, or uniformly using response cumulated in the whole trial, and found no significant difference in temporal performance between these two methods. The chosen time bin for training in Fig. 4 is selected at the period of 40–250 ms after stimulus onset and tested on MUA response averaged in serial 50 ms time bin from −50 to 250 ms relative to stimulus onset.

### Location decoding

We built a logistic regression model to decode each stimulus’s location (edge or surface), with the same population responses for the luminance decoder (see Supplementary information). The population response matrix is X, the same as the matrix used in luminance decoding. The units in the X are either all edge response or all surface response. The logistic regression model to fit population response to the square location is as follows:

10 $$P\left({{{{{\rm{location}}}}}}\; {{{{{\rm{is}}}}}}\; {{{{{\rm{edge}}}}}}{|X}\right)=\frac{1}{1+{e}^{X{{{{{\bf{W}}}}}}+w0}}$$

Here, W is an N by 1 vector of weights. When *p* ≥ 0.5 the population are predicted to be driven by stimulus edge, when *p* < 0.5 the population are predicted to be driven by stimulus surface. We trained the decoder by optimizing the weight matrix (W) to minimize the loss function (Eq. 11).

11 $$J=-\frac{1}{m}\left(\mathop{\sum }\limits_{i=1}^{m}({Y}_{i}{{{{{\rm{ln}}}}}}\left(P\right)+(1-{Y}_{i}){{{{{\rm{ln}}}}}}(1-P))\right)+\frac{\lambda }{2m}\left|{{{{{\bf{W}}}}}}\right|$$

Here, Y is an M by 1 vector representing the location of the stimulus, with 1 being edge and 0 being surface. We used an L1 regularization term to minimize the obtained weights and prevent overfitting. We randomly separated the dataset into a training set (60%), a validation set (15%) and a test set (25%). The regularization parameter (*λ*) was chosen as the one that gave the lowest MSE in a cross-validation test among 20 lambda values equally spaced (logarithmically) between 0.001 and 40. We used the ﻿fminunc function in Matlab to train the decoder. The results shown in this paper (Supplementary Fig. 6) are based on the regularization parameter at 0.1 for all sites.

### Model fitting and evaluation

We designed signal transmission models to simulate cortical processing for surface and edge response at the V1 output layer. We build models to fit response time courses of the output layer with data of the input layer and parameterized signal transmission procedure. One model is based on feedforward projections from the input layer (Fig. 6a, the upper panel). The predicted surface or edge response at the output layer is as follow:

12 $${R}_{{{{{{{\mathrm{outputlayer}}}}}}}}\left(i,\tau \right)=E\left(i,\tau \right)$$

The dynamic neural response depends on excitation input, *E*(*i*,*τ*), *i* is the location of the RF center of the neuron. The excitatory inputs are driven by feedforward input,

13 $$E\left(i,:\right)={E}_{{{{{{{\mathrm{feedforward}}}}}}}}\left(i,:\right)\otimes {{{{{{\mathrm{Kernel}}}}}}}_{E,i}\times {w}_{i}^{E}$$

14 $${E}_{{{{{{{\mathrm{feedforward}}}}}}}}\left(i,\tau \right)=\left(\mathop{\sum }\limits_{j=1}^{{{{{{\rm{end}}}}}}}{R}_{{{{{{{\mathrm{inputlayer}}}}}}}}\left(j,\tau \right)\times {{{\exp }}}^{\left(\frac{-{\left(j-i\right)}^{2}}{2\times {\sigma }^{2}}\right)}\right)$$

Here the feedforward excitation is a weighted summation of MUA in layer 4Ca and layer 4Cb, ${R}_{{{{{{{\mathrm{inputlayer}}}}}}}}={R}_{L4C\alpha }\times {a}_{i}+{R}_{L4C\beta }\times {b}_{i}$. The pooling rule of feedforward excitation is simulated as a gaussian function centered at the location *i*, the sigma of gaussian function predicted the pooling range. Parameter w is the gain of signal, which can be different between surface and edge signal. Kernels in the function indicated a signal transfer function, defined as:

15 $${{{{{{\mathrm{Kernel}}}}}}}_{k,i}={{{\exp }}}^{\left(\frac{-{\left({{\log }}\left(t\right)-{{{{{{\mathrm{delay}}}}}}}_{k,i}\right)}^{2}}{2\times {{{{{{{\mathrm{sigma}}}}}}}}_{k,i}^{2}}\right)}$$

The *k* represents the connection types contain *E, I* (*E* refer to excitation, *I* refer to inhibition, model A only has type *E*).

The other model is an interactive signal processing model (Fig. 6a, the lower panel). This model added inhibitory connections in the output layer. In this model, the excitatory feedforward inputs are combined with intralaminar inhibitory modulation:

16 $${R}_{{{{{{{\mathrm{outputlayer}}}}}}}}\left(i,\tau \right)=E\left(i,\tau \right)-I(i,\tau )$$

17 $$I\left(i,:\right)=\,{I}_{{{{{{{\mathrm{intralaminar}}}}}}}}\left(i,:\right)\otimes {{{{{{{\mathrm{Kernel}}}}}}}}_{I,i}\times {w}_{i}^{I}$$

The strength of horizontal modulation is simulated as a gaussian function centered at location *i*; the sigma of gaussian function predicted the range of horizontal influence.

18 $${I}_{{{{{{{\mathrm{intralaminar}}}}}}}}\left(i,\tau \right)=\left(\mathop{\sum }\limits_{j=1}^{{{{{{\rm{end}}}}}}}{R}_{{{{{{{\mathrm{inputlayer}}}}}}}}\left(j,\tau \right)\times {{{\exp }}}^{\left(\frac{-{\left(j-i\right)}^{2}}{2\times {\sigma }^{2}}\right)}\right)$$

The goodness of fit is evaluated as:

19 $${{{{{\rm{goodness}}}}}}\; {{{{{\rm{of}}}}}}\; {{{{{\rm{fit}}}}}}=1-\left(\frac{2* \mathop{\sum }\nolimits_{\tau =1}^{T}{\left({R}_{{{{{{{\mathrm{data}}}}}}}}\left(\tau \right)-{R}_{{{{{{{\mathrm{fit}}}}}}}}\left(\tau \right)\right)}^{2}}{T* \left({{{{{\rm{var}}}}}}\left({R}_{{{{{{{\mathrm{data}}}}}}}}\right)+{{{{{\rm{var}}}}}}\left({R}_{{{{{{{\mathrm{fit}}}}}}}}\right)\right)}\,\right)* \frac{T-1}{T-{pn}}$$

pn is the number of free parameters. We optimized model parameters to minimize the mean square error (MSE) between the estimated response time course and the actual response time course of the output layer, with fmincon functions written in matlab.

### Simulation

We first estimate the parameters in Eqs. (10–14) with 300 ms flashed square data and simulated two groups of data associated with different cortical processing with the obtained parameters and neuronal responses in the input layer. One simulation includes only feedforward excitation, and another consists of both feedforward and intralaminar inhibition. Then we added trial-by-trial noise on the simulated data sets to get population response consisting of numerous single trials. The noise we added is a Gaussian-distributed random variable that is independent across trials and with a standard deviation consistent with that of the real data.

### Statistics

To test the statistical significance of the difference between two groups of data we performed two-sample paired *t*-tests (e.g., S/E ratio between two periods of time, fitting goodness of two models) and unpaired *t*-tests (e.g., compare luminance decoding accuracy between different layers). To test whether filling-in indexes in multiple layers were significantly different, we performed one-way ANOVA for the multiple comparisons (see Supplementary Fig. 3). In order to confirm that the difference in decoding accuracy was stable, we used a bootstrap method during data training. We randomly selected N sites from all sites for decoding training, and repeated such operation several times. The multiple results are then subjected to paired or unpaired *t*-tests. To test the correlation between two groups of data we performed Pearson correlation. All error bars and measures of dispersion represent mean ± s.e.m unless indicated otherwise. All statistical tests are paired two-sided *t*-tests, except where noted.

### Reporting summary

Further information on research design is available in the Nature Research Reporting Summary linked to this article.

## Supplementary information

Supplementary Information

Reporting Summary

## Data Availability

Source data for Figs. 1–7 and Supplementary Figures are provided as a source data file. The dataset underlying the results described in our manuscript can be found in https://github.com/aileenyangyi/surface-luminance-coding-in-V1. Source data are provided with this paper.

## Source data

Source data

## Code availability

Custom MATLAB functions and scripts used to produce the results presented in this study are publicly available via GitHub: https://github.com/aileenyangyi/surface-luminance-coding-in-V1.
