# Supplementary material for: Coding strategy for surface luminance switches in the primary visual cortex of the awake monkey
Source: Nat Commun. 2022 Jan 12;13:286. doi: 10.1038/s41467-021-27892-3 (PMC8755737; doi:10.1038/s41467-021-27892-3)
Supplement: Supplementary file 1 — Supplementary Information [file 41467_2021_27892_MOESM1_ESM.docx]

**Coding strategy for surface luminance switches in the primary visual cortex of the awake monkey**

**Supplementary Information**

**Supplementary Notes**

**Reading of surface luminance requires location information**

To examine whether the stimulus luminance could be read out accurately without knowing the location type of input response, we trained a decoder for luminance that treated each site's edge response and surface response exactly alike. As a result, the weight matrix (W) in luminance decoding model (equation 1) is the same for surface response (X_surface_) and edge response (X_edge_) of the same group of cells.

*L* = *X*W+*w0* (1)

It turns out that the performance of such a decoder is much worse than the decoder with a different weight matrix for edge response and surface response (Figure S5, black curve). This result suggests that the optimal way to interpret luminance information is to treat surface and edge response differently, and the visual system can identify input types (location) besides luminance.

Next, we asked whether the neural population can provide information for edge and surface locations to downstream sites. We build a location decoder (logistic regression) to tell whether a group of neurons is driven by a stimulus’ surface or edge (Figure S6A, see details in ‘**Location decoder’ below**). We found that the downstream site can correctly identify whether population inputs are driven by a stimulus surface or its edge (Figure S6B, black curve). Interestingly, the latency for identifying a stimulus’ location is faster than that for identifying a stimulus’ luminance (Figure S6B, insets), suggesting that location information might be used to guide the decoding of luminance.

Since edge-driven responses are always stronger than surface-driven responses, the location decoder may simply classify response type through averaged response amplitude. In that way, the decoder may not differentiate between responses driven by a surface with high luminance and responses driven by an edge with lower contrast. To ensure the decoder could identify the location of stimuli at different luminance levels, we simulated a set of population responses to different luminance levels by rescaling the mean response amplitude while keeping the structure of population responses (Figure S6C, upper panels). We found that the downstream site (the decode) was still able to correctly distinguish population responses triggered by different stimulus locations, even under the circumstances that the average responses of the two types of responses were similar (Figure S6C, lower panels).

Finally, we tested whether the downstream neurons can correctly identify luminance information under the guidance of decoded location information (Figure S6D, upper, see details in ‘**Multiplex decoding procedure**’ below). The result shows that the decoder can correctly distinguish luminance levels between a bright surface and a low-contrast edge, even though these two stimuli stimulated similar levels of neuronal activities (Figure S6D, lower). Thus, our results suggest that a multiplexed decoding procedure might be true, and the downstream neurons might prioritize the reading of location information to guide the reading of luminance.

**Supplementary Methods**

**Location decoder:** We built a logistic regression model to decode each stimulus's location (edge or surface) with the same population responses for the luminance decoder (see Supplementary information). The population response matrix is X, the same as the matrix used in luminance decoding. The units in the X are either all edge response or all surface response. The logistic regression model to fit population response to the square location is as follows:

$P\left( location is edge|X \right)= \frac{1}{1+e^{X\mathbf{W}+w0}}$ (2)

Here, W is an N by 1 vector of weights. When p≥0.5, the population is predicted to be driven by stimulus edge; when p<0.5, the population is predicted to be driven by stimulus surface. We trained the decoder by optimizing the weight matrix (W) to minimize the loss function (equation 3).

$J= -\frac{1}{m}\left( \sum_{i=1}^{m} (Y_{i}\ln\left( P \right)+(1-Y_{i})ln(1-P)) \right)+\frac{\lambda}{2m}\left| \mathbf{W} \right|$ (3)

Here, Y is an M by 1 vector representing the location of the stimulus, with 1 is edge and 0 is surface. We used an L1 regularization term to minimize the obtained weights and prevent overfitting. We randomly separated the data set into a training set (60%), a validation set (15%) and a test set (25%). The regularization parameter (λ) was chosen as the one that gave the lowest MSE in cross-validation test among 20 lambda values equally spaced (logarithmically) between 0.001 and 40. We used fminunc function in Matlab to train the decoder. The results shown in this paper (Fig. S6) are based on regularization parameter at 0.1 for all sites.

**Multiplex decoding procedure:** The multiplexed model is a successive combination of a location decoder and two luminance decoders (for surface or edge responses). The three decoders were trained separately in the same way described before (see ‘location decoder’ described above and ‘luminance decoding’ in the main text).

In interpreting surface luminance information for the testing dataset, the location decoder identifies whether the neuronal population is driven by a stimulus’ surface or edge (step 1). Then the location information is used to guide luminance decoding: a surface location will lead the population responses to one luminance decoder (W_surface_), otherwise the other luminance decoder (W_edge_) will be used (step 2). Finally, a single luminance value is read out by the chosen luminance decoder (step 3).

**Supplementary Figures**

**Supplementary Figure 1: Distribution location and size of recording sites’ receptive fields (RF)**

A. Receptive field size mapping of an L3 site.

B. Spatial distribution of receptive fields. Circles depict the location and size of CFRs. Blue dashed lines marked eccentricity at 1 degree and 5 degree.

C. Distribution of classical receptive field (CRF) sizes (total n = 630). The CRF is fitted with a two-dimensional Gaussian function (red curve). CRF size is estimated as four times of gaussian sigma.

D. Distribution of extra classical receptive field (eCRF) sizes (total n = 90). The eCRF size is measured with response tuning to different grating sizes. Inset shows the cumulative distribution of eCRF size. Left: size tuning of an example site, errorbars show ± s.e.m. across response repeats, n = 20.

Source data are provided as a Source Data file.

**Supplementary Figure 2: Cortical presentation of black surface and edge in V1 layers**

A. Population averaged spatial-temporal responses (MUA) to different positions of a black square at different layers.

B. Laminar pattern of the neuronal response to stimulus edge (left) and surface (right).

C. Ratio of averaged surface response to averaged edge response (S/E ratio) at different cortical depth. The S/E ratio is smoothed at each step (0.02 depth units) with a window at 0.08 depth units. Solid curve and shading represent mean ± s.e.m.. Total number of sites is 630.

Source data are provided as a Source Data file.

**Supplementary Figure 3: Filling-in index of surface responses across V1 layers and their correlations**

A. Filling-in index of response to the white surface at different cortical depths. The filling-in index is smoothed in depth (between nearest 0.08 depth units); Solid curve and shading represent mean± s.e.m. (n = 507).

B. Averaged filling-index at different V1 layers. Bars represent mean filling-in index across sites (± s.e.m.), with individual data superimposed (n = 63, 63, 44, 77, 68, 91, 101 sites in layers from L2 to L6 respectively). There was no significant difference in filling-in index among layers. p = 0.9126, F(6,500) = 0.35, one-way ANOVA without adjustment.

C. Pearson’s correlation of filling-in strength among simultaneously recorded sites at different V1 layers. p <0.05 except the one marked with n.s. for p = 0.091.

Terms in D-F are the same as A-C but for responses to a black surface. There was significant difference in filling-in index among layers. p = 4.705e-17, F(6,572) = 16.01, one-way ANOVA without adjustment.

Source data are provided as a Source Data file.

**Supplementary Figure 4: Filling-in strength and surface response sustainability are highly correlated**

A. Correlation between filling-in strength and response sustainability at the output layer (left for edge response and right for surface response). Sustainability is defined as a ratio of the integrated response at the later time to the integrated response during the whole time. r marks Pearson’s correlation coefficient with corresponding p value.

B. Similar to A but for the input layer.

C. Pearson’s correlation between filling-in strength and sustainability of edge response and surface response at different layers. *p = 0.0224, ***p< 1.0e-5 for the significance of correlations.

Source data are provided as a Source Data file.

**
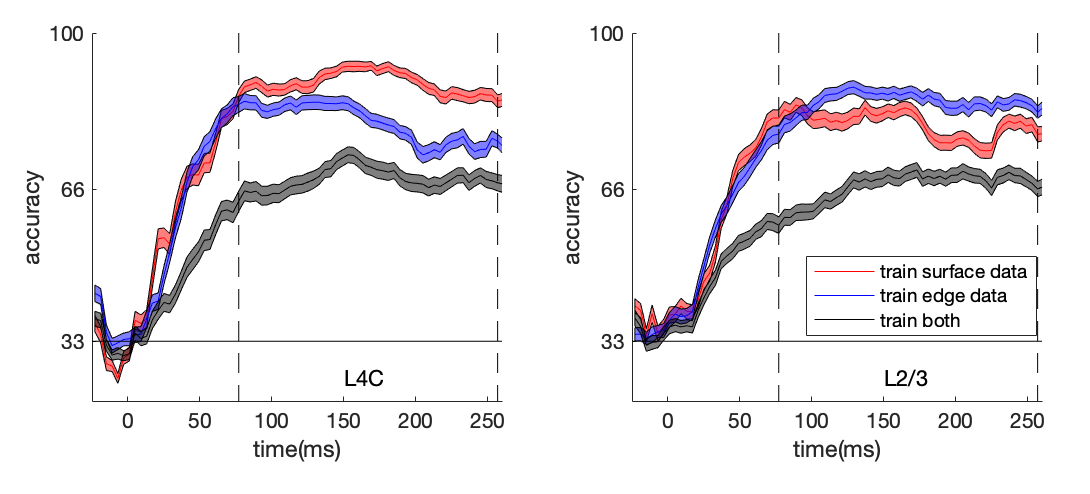
**

**Supplementary Figure 5: Performance of luminance decoding gets worse if the decoder does not distinguish edge and surface responses**

Temporal development of decoding performances in interpreting luminance through three strategies (left: input layer; right: output layer). The black curve represents the average decoding performance when the edge and surface responses are used indiscriminately for decoding luminance. The red and blue curves represent the average decoding performance of the surface or boundary responses, respectively. Solid curve and shading represent mean ±s.e.m. across populations, n = 20.

Source data are provided as a Source Data file.

**Supplementary Figure 6: Performance of location decoding**

A. Demonstration of location decoder with same population responses used by luminance decoder.

B. Temporal development of performance for decoding stimulus location (black) and decoding stimulus luminance (red and blue). The performance accuracy for decoding luminance was rescaled to match the baseline level for decoding stimulus locations. The insets show the latencies for luminance decoding and location decoding, which were defined as the time when decoding accuracy reach 70%. Bars present the mean latency across populations (±s.e.m.), with individual data superimposed (n=100). ***p<0.001, two-sided independent t-test.

C. Upper panels show simulated population response to stimulus edge and surface at different luminance levels. Lower panels show the discrimination ability between edge and surface at different luminance levels for stimuli. Solid curves and shadings represent mean±s.e.m. across sites (upper panels, n = 71) or which across populations (low panels, n =100).

D. Performance of a decoder for luminance discrimination guided by location information decoded by the same population responses. Upper panel shows the framework of a ‘multiplex decoder’ with the ability to decode both luminance and location information; lower left panel shows the performance of the multiplex decoder at different luminance levels based on population responses at different locations (edge or surface). Bars present mean prediction across population (± s.d.), with individual data superimposed (n = 100); ***p<0.001, tow-sided independent t-test. lower right panel shows population activity to stimuli corresponding to those in the left panel. There is no significant difference between neural activities: White condition, n.s.: p = 0.569; Black condition, n.s.: p = 0.784; tow-sided independent t-test. Bars present mean activity across sites (± s.d.), with individual data superimposed (n = 71).

Source data are provided as a Source Data file.


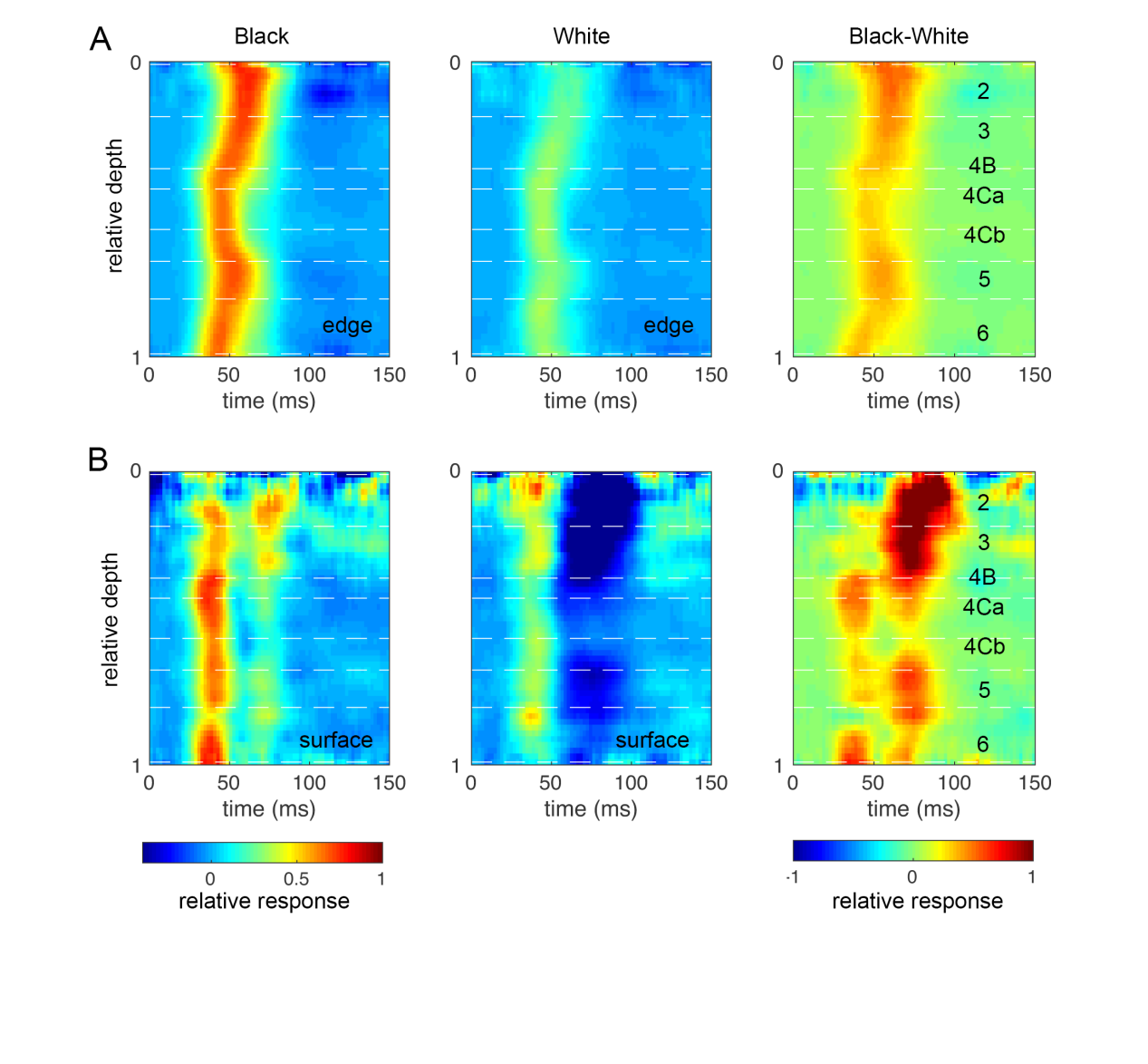


**Supplementary Figure 7: Laminar pattern of relative response to black and white edge and surface**

A. Normalized MUA to the edge of black or white square. Normalized responses are defined as: $R_{norm}={R/max(R}_{black\_edge}+R_{white\_edge})$

B. Normalized MUA to the surface of black or white square. Normalized responses are defined as: $R_{norm}={R/max(R}_{black\_surface}+R_{white\_surface})$


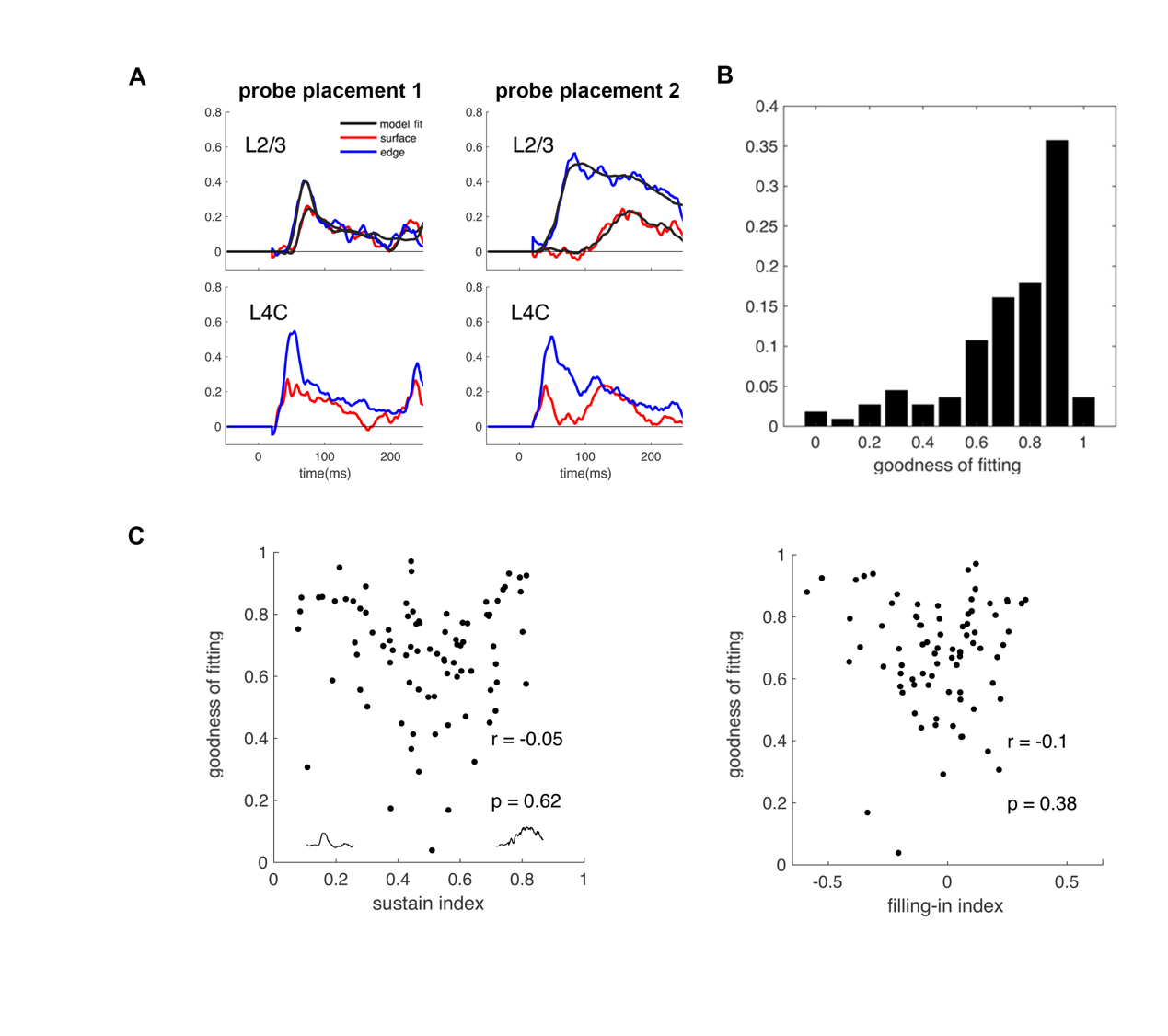


**Supplementary Figure8: Model B could well reconstruct responses in 300ms flashed square experiment**

A. Fitting performance of model B for two example probe placements. The lower panel depicted the response of the input layer, and the upper panel depicted the response of the output layer, red for surface and blue for edge. Black curves in upper panel show fitting results.

B. Distribution of fitting performance of model B for all the data in 300ms flashed square experiment.

C. Correlation between fitting performance and sustain index (left panel), between fitting performance and filling index. r marks Pearson’s correlation coefficient, with corresponding p value.

Source data are provided as a Source Data file.


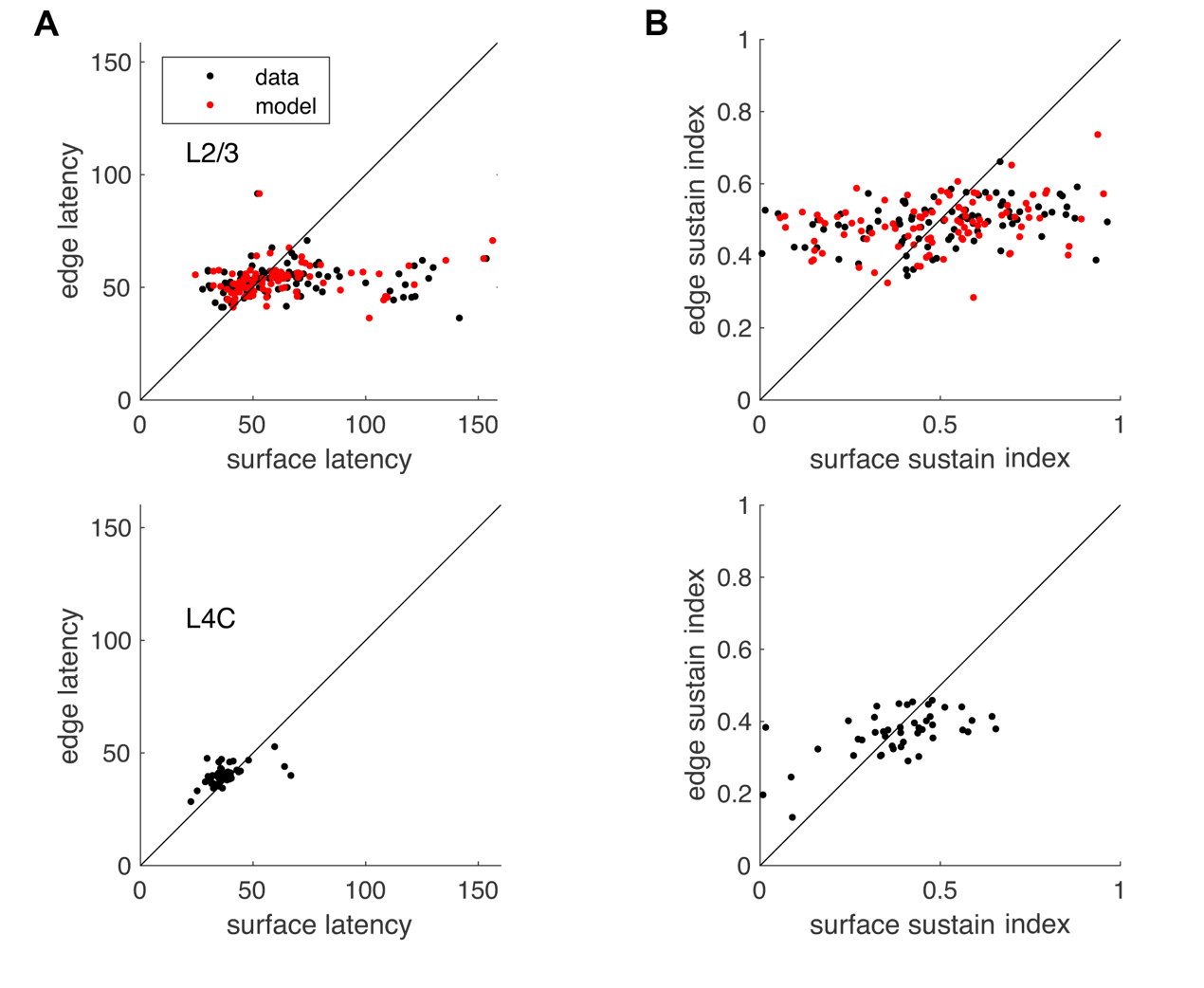
­

**Supplementary Figure 9: Model B captures the altered surface response latency and sustainability in the output layer.**

The lower panel shows the dynamic characteristics (A for response latency and B for sustain index) of response in the input layer; the upper panel shows the dynamic characteristics of response in the output layer. The black dots represent features of the recorded data, and the red dots represent model predictions.

Source data are provided as a Source Data file.

**
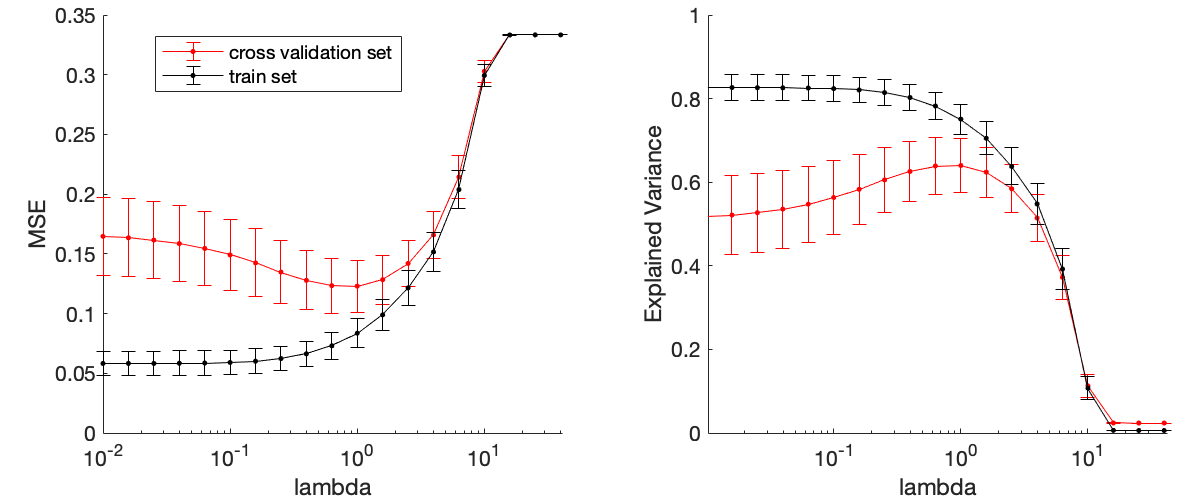
**

**Supplementary Figure 10. The performance of the decoder with different regularization parameters (lambda) for training procedure.**

Left, the mean square errors of the training set (black curve) and the cross validation set (red curve) as a function of lambda. Right, the explained variance of the training set (black curve) and the cross validation set (red curve) as a function of lambda. Curves and error bars present mean$\pm$s.d. across populations, n = 20.

Source data are provided as a Source Data file.

**
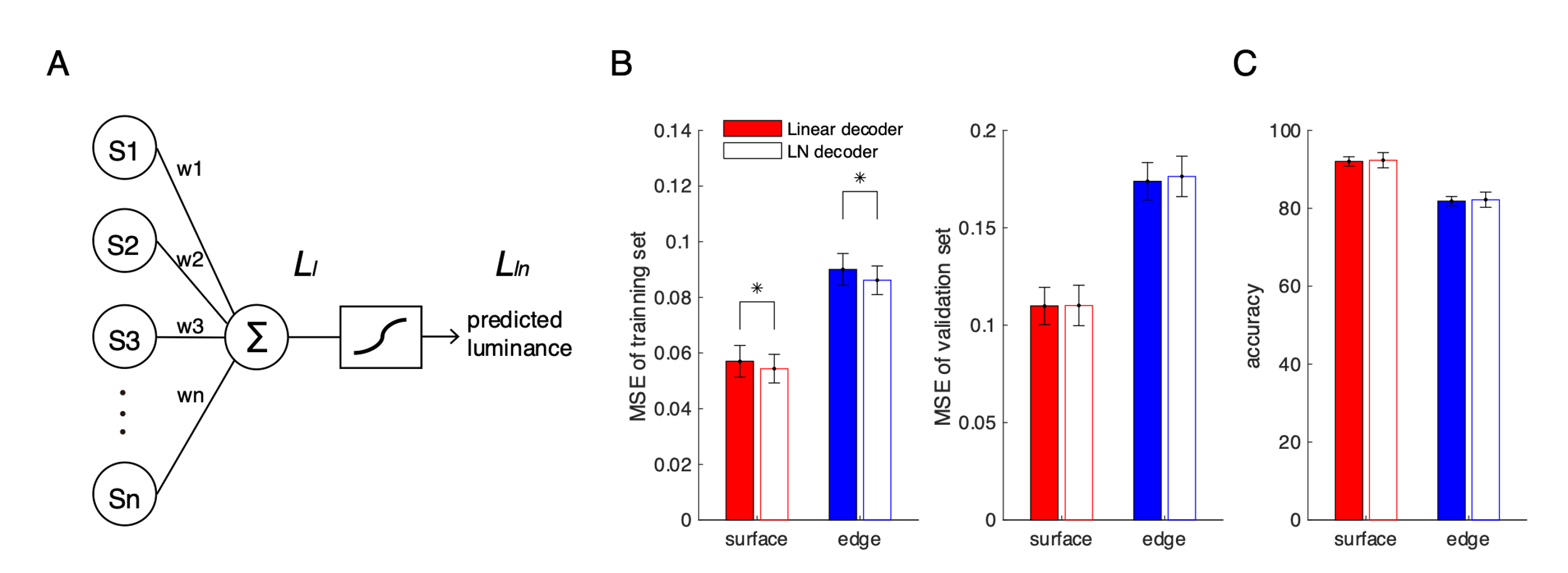
**

**Supplementary Figure 11. The performance of a LN decoder compared to that of a linear decoder**

A. Framework of the LN decoder. The LN decoder predicts the stimulus's luminance with a weighted summation and a nonlinear transformation of population MUA response.

B. The averaged mean square errors (MSE) of the training set (left) and the validation set (right) using linear model and LN model.

C. Decoding accuracy for luminance by the linear model and LN model.

Bars in b and c represent mean values across populations (± s.e.m.).

Source data are provided as a Source Data file.

**Supplementary Figure 12. Interlaminar and intralaminar correlations between neural activity and inhibitory components.**

A. Relationship between estimated inhibition in L2/3 and neural response measured in L4C. Pearson’s correlation coefficient is 0.52.

B. Relationship between estimated inhibition in L2/3 and neural response measured in L2/3. Pearson’s correlation coefficient is 0.3.

r in A and B marks Pearson’s correlation coefficient, accompanied by its corresponding p value.

Source data are provided as a Source Data file.
